# Letter from Hugh J Jewett

**DOI:** 10.4103/0970-1591.57908

**Published:** 2009

**Authors:** Ganesh Gopalakrishnan

**Affiliations:** Christian Medical College, Dr. Ida Scudder Road, Vellore - 632 004, Tamilnadu, India

Dear Editor,

Carcinoma prostate is the most common urological cancer that has attracted worldwide debate. Urologists, radiation oncologists, epidemiologists, physicians, lay public and celebrities have all joined in the discussion.

As urologists, we avidly support radical prostatectomy as the best treatment modality to cure this cancer.

As urologists, we have probably forgotten that radical prostatectomy is not a new operation. Hugh Hampton Young performed the first radical perineal prostatectomy in 1904.

As a resident, in my quest to understand the nuances of appropriate urological terminology, I began to wonder as to why this operation was termed “radical” perineal prostatectomy.

As general surgeons, we were taught that the term “radical” is used in oncological surgery when the surgical exercise involves lymph node clearance. This is not the case in radical perineal prostatectomy.

In 1977, I wrote to Hugh J Jewett for an answer. I thought it appropriate to share this letter (verbatim) as it was written to me. You might ask why this letter is so late. To be honest, I was looking for it for a long time and found it while I was clearing my office on the eve of my retirement from the Urology Department of Christian Medical College, Vellore.

